# Molecular Marker Discovery and Evaluation: *EGF* rs1897990 and rs1524106 Variants in a China Lung Adenocarcinoma Young Population

**DOI:** 10.1111/crj.70113

**Published:** 2025-07-22

**Authors:** Huiwen Pan, Xiangyang Wang, Lijie Zheng, Jinye Wang, Guowen Ding, Jingfeng Zhu, Zhijie Fang

**Affiliations:** ^1^ The Affiliated People's Hospital of Jiangsu University Zhenjiang Jiangsu China; ^2^ Troop 73071 of the PLA Xuzhou Jiangsu China; ^3^ Department of Otolaryngology The Affiliated Suzhou Hospital of Nanjing Medical University, Suzhou Municipal Hospital, Gusu School, Nanjing Medical University Suzhou China

**Keywords:** *EGF*, lung adenocarcinoma, rs1524106, rs1897990, SNP, young patients

## Abstract

**Objective:**

The objective of this study is to investigate the genetic susceptibility and risk factors of *EGF* gene rs1897990 and rs1524106 in lung adenocarcinoma young patients aged ≤ 45 years.

**Methods:**

A case–control study was conducted. DNA was extracted and identified from 88 samples from case and control groups by single‐nucleotide polymorphism assay. PCR amplification was performed by TaqMan probe method, and the factors of smoking, drinking, sex, and age were also included. To investigate the clinical factors and genotyping differences between case and control groups.

**Results:**

Smoking was an influential factor in young lung adenocarcinoma patients. The mutation frequency of *EGF* gene rs1897990 CT heterozygous mutant was different between the two groups (*p* = 0.021). The T alleles of *EGF* rs1897990 and rs1524106 were significantly different between the two groups (*p* = 0.034 and *p* = 0.023).

**Conclusions:**

The young lung adenocarcinoma population (≤ 45 years old) is susceptible to *EGF* rs1897990 and rs1524106 variants, with smoking being another risk factor. Additionally, smoking may enhance the risk of *EGF* rs1897990 and rs1524106 variants threatening the development of lung adenocarcinoma.

## Graphical abstract

The core EGF gene variants in string interaction network rs1897990 and rs1524106 are significant risk factors for lung adenocarcinoma in young individuals, particularly when combined with smoking. Tobacco use appears to potentiate the oncogenic effects of these variants, further increasing susceptibility to early‐onset lung adenocarcinoma.

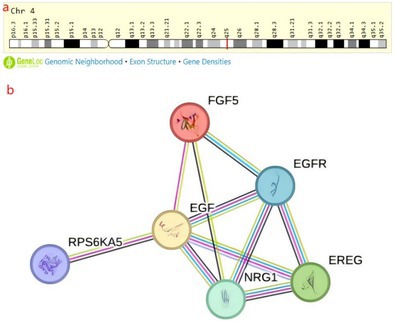

## 1 Introduction

Lung cancer remains one of the leading causes of cancer‐related diagnoses and deaths in China [1], with an increasing trend in the number of young patients. Compared to the previously predominant squamous cell carcinoma, lung adenocarcinoma has become the most common pathological type of lung cancer [2]. Tumor invasion and metastasis represent an extremely complex process regulated by multiple genes and involving several developmental steps. This process entails abnormalities in the structure and function of a series of related genes, with tumor angiogenesis considered a crucial mechanism for tumor invasion and metastasis. Vascular endothelial growth factor (*VEGF*) and epidermal growth factor receptor (*EGFR*) play key roles in promoting endothelial cell division and proliferation, as well as in facilitating angiogenesis. A bioinformatics analysis indicated that in female patients with non‐small cell lung cancer (NSCLC), *EGF* may be an important gene in the mechanisms of NSCLC onset and prognosis, representing a potential therapeutic target [3].

The *EGF* gene is located on human chromosome 4 (4q25) and encodes an *EGF* protein that regulates cell proliferation, differentiation, migration, and survival by binding to the epidermal growth factor receptor (*EGFR*) [4, 5]. Rs1897990 is a single‐nucleotide polymorphism (SNP) located in the promoter region of the human epidermal growth factor (*EGF*) gene (5′‐untranslated region, 5′‐UTR), which manifests itself as a C/T base variant. This locus is located in a key region of gene regulation and may regulate the transcriptional activity of the *EGF* gene by affecting transcription factor binding capacity or chromatin accessibility, which in turn affects the expression level of *EGF* protein [6, 7]. Rs1524106 is a synonymous SNP located in the exon region (coding region) of the *EGF* gene, which manifests as a C/T(G/A) base variation with no alteration of the encoded amino acid (arginine) [8].

The T allele at rs1897990 upregulates epidermal growth factor expression by enhancing promoter activity, which in turn activates *EGF*R downstream signalling pathways (e.g., *MAPK/ERK*), promotes cell proliferation, and inhibits apoptosis [9]. In lung adenocarcinoma studies in the Chinese population, carriers of the rs1897990‐T allele had a significantly increased risk (OR = 1.32, 95% CI: 1.08–1.60), possibly related to aberrant activation of *EGFR* signalling driven by *EGF* overexpression in the tumor microenvironment [10]. In addition, studies linking this locus with gastric cancer risk have shown that the T allele increases gastric cancer susceptibility by regulating *EGF*‐mediated inflammatory responses and mucosal repair disorders [11]. However, some studies did not find a significant association with lung cancer, such as a European cohort analysis that showed no statistically significant polymorphism of rs1897990 [12], suggesting that the population's genetic background or environmental exposures (e.g., smoking) may influence its effects. Functional experiments further confirmed that the rs1897990‐T allele could enhance promoter reporter activity in lung cancer cell lines and potentially link it with resistance to *EGFR*‐targeted therapy [13].

Although synonymous, it may indirectly regulate *EGF* protein expression by affecting mRNA secondary structure stability, splicing efficiency, or translation rate. Research abroad has shown that young lung cancer patients at initial diagnosis often present with later stages and more severe conditions [14, 15]. It is worth exploring whether such cases are related to abnormalities in the *EGF* gene. This study aims to identify two representative *EGF* gene loci through predictive modeling methods and analyze their distribution in young adenocarcinoma patients. Additionally, by considering smoking—one of the most common etiological factors of lung cancer—this research further investigates the association between young lung adenocarcinoma and *EGF* gene polymorphisms, as well as environmental factors.

## 2 Materials and Methods

### 2.1 Study Subjects

A total of 43 young lung adenocarcinoma patients (aged ≤ 45 years, defined as young lung adenocarcinoma patients) were collected from the Department of Thoracic Surgery at the People's Hospital Affiliated with Jiangsu University between January 2010 and June 2023, serving as the case group. The ethical approval number is SQK‐20220167‐Y. Ethics statement: The present study was approved by the Ethics Committee of the Affiliated People's Hospital of Jiangsu University. Written informed consent was obtained from patients. The specimens were obtained from surgically resected lung adenocarcinoma tissues, specifically the remaining tissue blocks after pathological examination. The control group consisted of 45 healthy individuals randomly selected from the health examination center, all of whom had no prior history of tumors or familial genetic diseases. There were no statistically significant differences in gender and age between the two groups, and the individuals in both groups were not related by blood.

The average age of the overall sample was 40 ± 4.93 years. All participants were of Han Chinese ethnicity. Lung adenocarcinoma patients were clinically and pathologically diagnosed, and all cases were primary tumors without prior radiotherapy or chemotherapy, excluding any secondary or recurrent tumors and other malignancies.

This study was approved by the Ethics Review Committee of Jiangsu University and the People's Hospital Affiliated to Jiangsu University. The purpose and significance of the study were explained to the participants, and informed consent was obtained from all patients.

### 2.2 Experimental Methods

The minimum allele of rs1897990 in case group is T and the MAF value is 0.38 The minimum allele of rs1524106 is T, and the MAF value is 0.43. Clinical factors such as gender, age, smoking and drinking habits, pathological grading, and staging were recorded for both the case and control groups using a combination of electronic medical records and Excel spreadsheets. SNP genotyping experiments: 1.0 mL of EDTA anticoagulated whole blood was collected from all study subjects after clinical examination to extract DNA. The extraction process followed the steps outlined in the kit (Tiangen, RT405‐13, DP318‐03). After DNA extraction, primers and probes were designed, followed by PCR amplification and fluorescence signal detection using the TaqMan probe method to assess gene polymorphism. We used a blood DNA kit method to extract the DNA completed and PCR test according to the sequence table designed by primers and the preparation steps of PCR mixed reaction solution. Reaction procedure: predenaturation 95°C, 5 min, 1‐cycle number, denaturing 95°C, 15 s, 40 cycles, annealing/elongation 60°C, 30 s, 1‐cycle number. Finally, the SNPs polymorphism was identified by the sequencer. SNPs were genotyped using TaqMan SNP Genotyping Assays (Thermo Fisher Scientific, USA) on a QuantStudio 5 Real‐Time PCR System (Applied Biosystems, Thermo Fisher Scientific, Singapore). Reactions were performed in 96‐well plates with TaqMan Universal PCR Master Mix (Thermo Fisher Scientific, Catalog No. 4304437).

After the extension reaction, the ABI3730XL was used for sequencing to detect genotyping. The gene polymorphism was detected by the Snapshot method, and the 5% samples were randomly selected for reinspection to ensure the accuracy of the test results.

## 3 Statistical Analysis

All statistical analyses were performed using SPSS statistical software (SPSS Inc., Chicago, Illinois, USA, version 20.0). Comparisons between groups were conducted using the *χ* ^2^ test. Logistic regression analysis was used to calculate the odds ratio (OR) and its 95% confidence interval (95% CI) and to adjust for confounding factors. Hardy–Weinberg equilibrium (HWE) analysis was conducted to assess genetic balance. Logistic regression analysis calculated the OR and 95% CI to analyze the relative risk of polymorphic loci and alleles. All statistical tests were two‐tailed, with a significance level set at *p* < 0.05.

Estimation steps for sample content. The following sample size calculations are based on the hypothesis that exposure factors (gene polymorphisms) are associated with lung adenocarcinoma, with OR (odds ratio) as the core parameter: 1. Determine the parametersα (significance level): set to 0.05 (two‐sided test). *β* (statistical power): usually set to 0.2 (i.e., power 1‐*β* = 80%).OR (Effect Size): The OR value of the two loci set is 1.10 according to the previous pre‐experiment and the reference of the literature. Control group exposure rate (*P0*): Reference exposure ratio of healthy people, as well as MAF values. Finally, the sample content estimation is carried out using Power Samples software. Because the prevalence of lung adenocarcinoma in young patients was very low, we included studies with small sample sizes.

## 4 Results

### 4.1 Baseline Data of Polymorphism at *EGF* Gene rs1897990 and rs1524106 Loci

**FIGURE 1:**
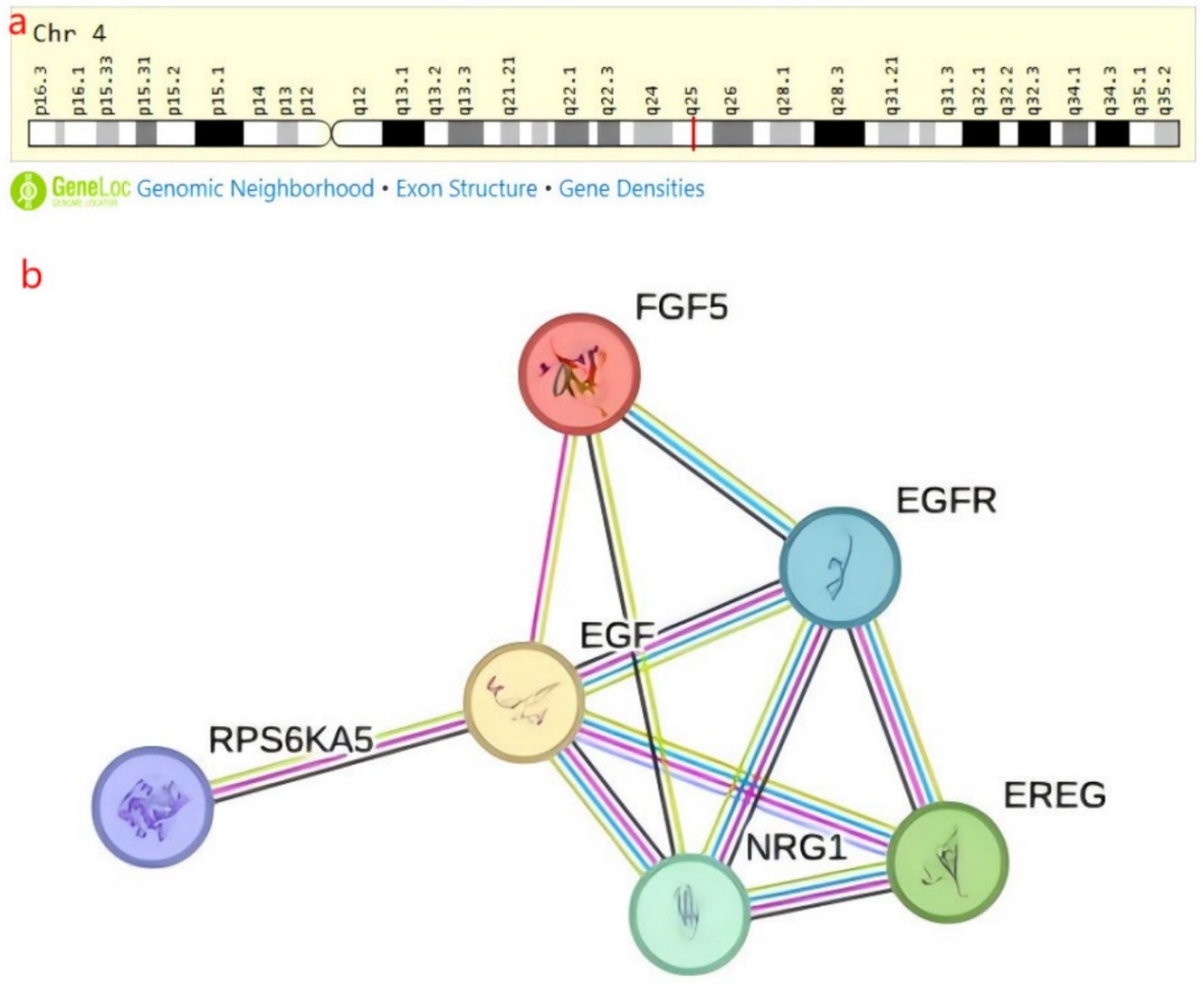
*EGF* gene in genomic location and string interaction network. (a) Bands according to Ensembl, locations according to gene location (and/or NCBI Gene and/or Ensembl if different). (b) String Interaction network, Preview showing top 5 string interactants (based on experimental score).

Primary information for *EGF* gene rs1897990 and rs1524106 polymorphisms was shown from Table 1. *EGF* gene rs1897990 and rs1524106 are both located on chromosome 4q25 (Figure 1). According to the genecards database consulted information, the gene two SNPs location segments are located at 8:63250311 and 7:22745364. The TaqMan method was used to type the genes locus, and all samples were successfully tested (100%), as detailed in Table 1.

**TABLE 1:** Primary information for *EGF* gene rs1897990, rs1524106 polymorphisms.

Genotyped SNPs	*EGF* rs1897990	*EGF* rs1524106
Chromosome	4	4
Alleles	C > T	T > A,G
Cytogenetic^a^	4q25	4q25
Location^a^	4: 109963299 (GRCh38)	4: 109940952 (GRCh38)
Genotyping method^b^	TaqMan	TaqMan
% Genotyping rate	100%	100%

^a^ http://www.genecards.org/.

^b^ LDR: ligation detection reaction.

The *EGF* gene polymorphism loci rs1897990 and rs1524106 are located on chromosome 4q25(Figure 1a). In the string interaction network figure, the *EGF* gene and several other genes are inextricably linked, such as *EGFR*, and they have biological effects with each other to maintain the stability of the network (Figure 1b).

### 4.2 Comparison of Variables and Risk Factors Between Lung Adenocarcinoma Cases and Controls

In Table 2, there was no statistically significant difference in age between the two groups (*p* > 0.05), indicating that the data for age in both groups are well matched and comparable. The proportion of male patients in the case group was slightly higher than that in the control group (53.49% vs. 44.44%); however, the difference between the two groups was not statistically significant (*p* = 0.396).

**TABLE 2:** Chi‐square test of subjects statistical variables and risk factors.

Variables	Case (*n* = 43)	Control (*n* = 45)	*p*
	*n*%	*n*%	
Age mean ± SD	41 ± 0.52	40 ± 1.76	> 0.05
Sex			
Male	23 (53.49)	20 (44.44)	
Female	20 (46.51)	25 (55.56)	0.396
Smoking			
Yes	14 (32.56)	1 (2.22)	
No	29 (67.44)	44 (97.78)	**< 0.001**
Alcohol			
Yes	6 (13.33)	2 (4.44)	
No	39 (86.67)	43 (95.56)	0.266

*Note:* *T‐test calculation values.

Among the young lung adenocarcinoma patient population, a history of smoking may be a contributing factor, as the chi‐square test comparison between the case and control groups showed statistical significance (*p* < 0.001).

The distribution frequency of alcohol consumption did not show a statistically significant difference between the two groups (*p* = 0.266), all above from Table 2.

Table 3 is the clinical data on the included population, such as gender, clinical stage, pathological stage, surgical procedure selection, and pathological type classification data. The detailed frequency and percentage of each group are recorded in the Table 3, total case sample is 43.

**TABLE 3:** Pathology and stage information table of the sample.

Factor	Type	Frequencies
Gender	Male	23 (53.49%)
	Female	20 (46.51%)
TNM stage	T1	33 (76.74%)
	T2	5 (11.63%)
	T3	4 (9.30%)
	T4	1 (2.33%)
	N0	26 (60.46%)
	NI	7 (16.28%)
	N2	10 (23.26%)
	M0	43 (100%)
Pathological stage	IA, IB	24 (55.8%)
	IIA, IIB	4 (9.30%)
	IIIA	13 (30.23%)
	IIIB, IV	2 (4.65%)
Surgical procedure	Minimally invasive	42 (97.67%)
Pathological type	Open surgery	1 (2.3%)
	Adenocarcinoma	43 (100%)

### 4.3 Comparison of Genotype Frequencies of *EGF* Gene rs1897990 and rs1524106 Between Case and Control Groups

#### 4.3.1 DNA Extraction and Assessment of Concentration and Purity

**FIGURE 2:**
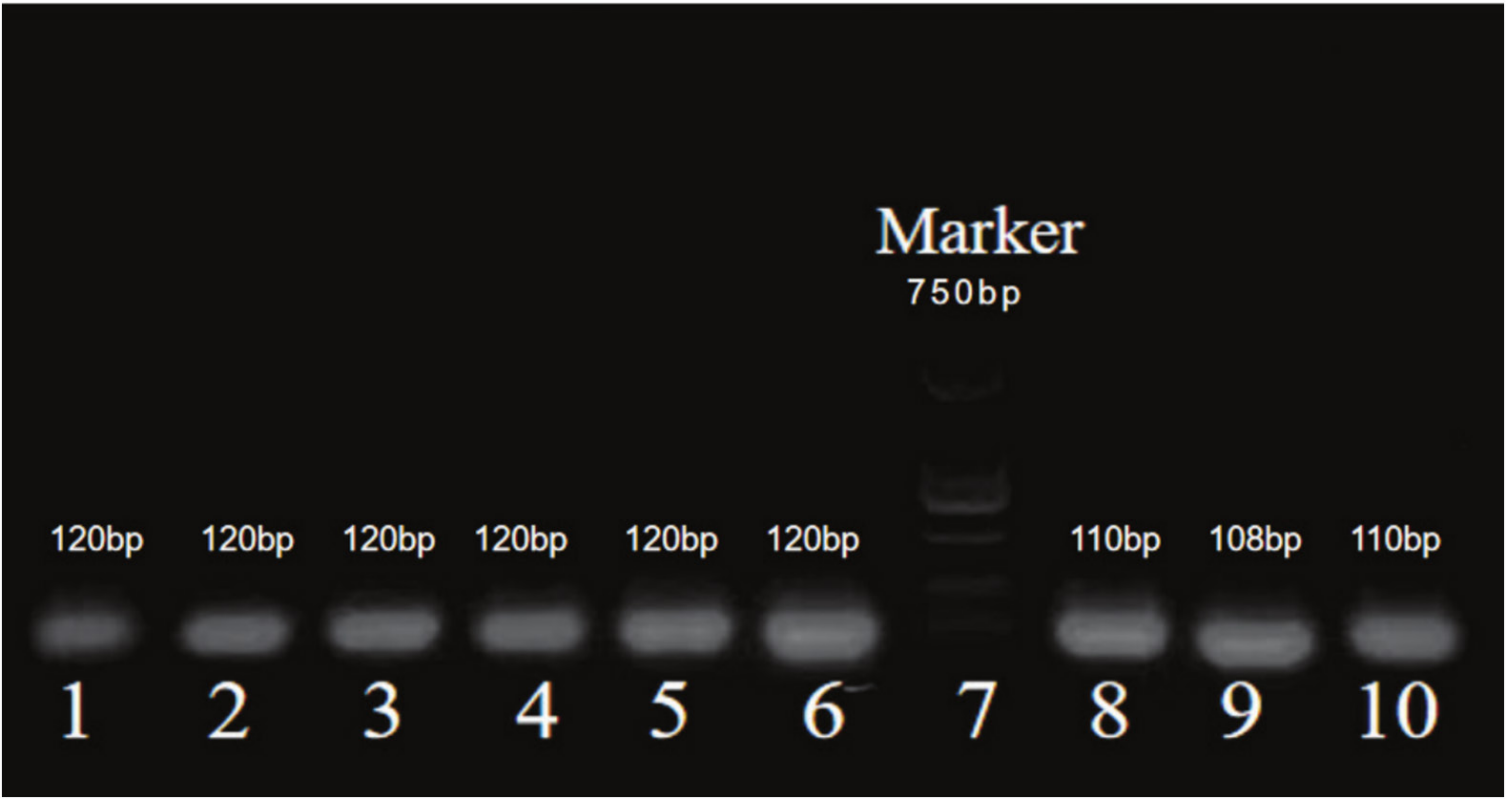
DNA concentration and purity identified by 2.5% agarose gel electrophoresis. Marker length:750 bp; 1–6: rs1897990,120 bp; 8–10: rs1524106,110 bp.

From Figure 2: DNA concentration and purity were assessed using 2.5% agarose gel electrophoresis. To ensure the reliability and accuracy of the results, each sample was run in triplicate or more.

The results showed that the electrophoresis DNA bands were clear, the lengths were distinctly layered, and the corresponding wells matched accurately. The total length of the Marker was 750 bp, the DNA fragment length at the rs1897990 locus was 120 bp, and the DNA fragment length at the rs1524106 locus was 110 bp, all of which met the quality control conditions for subsequent experiments.

#### 4.3.2 Identification of Polymorphisms at *EGF* Gene rs1897990 and rs1524106 Loci Through Gene Typing Sequencing

**FIGURE 3:**
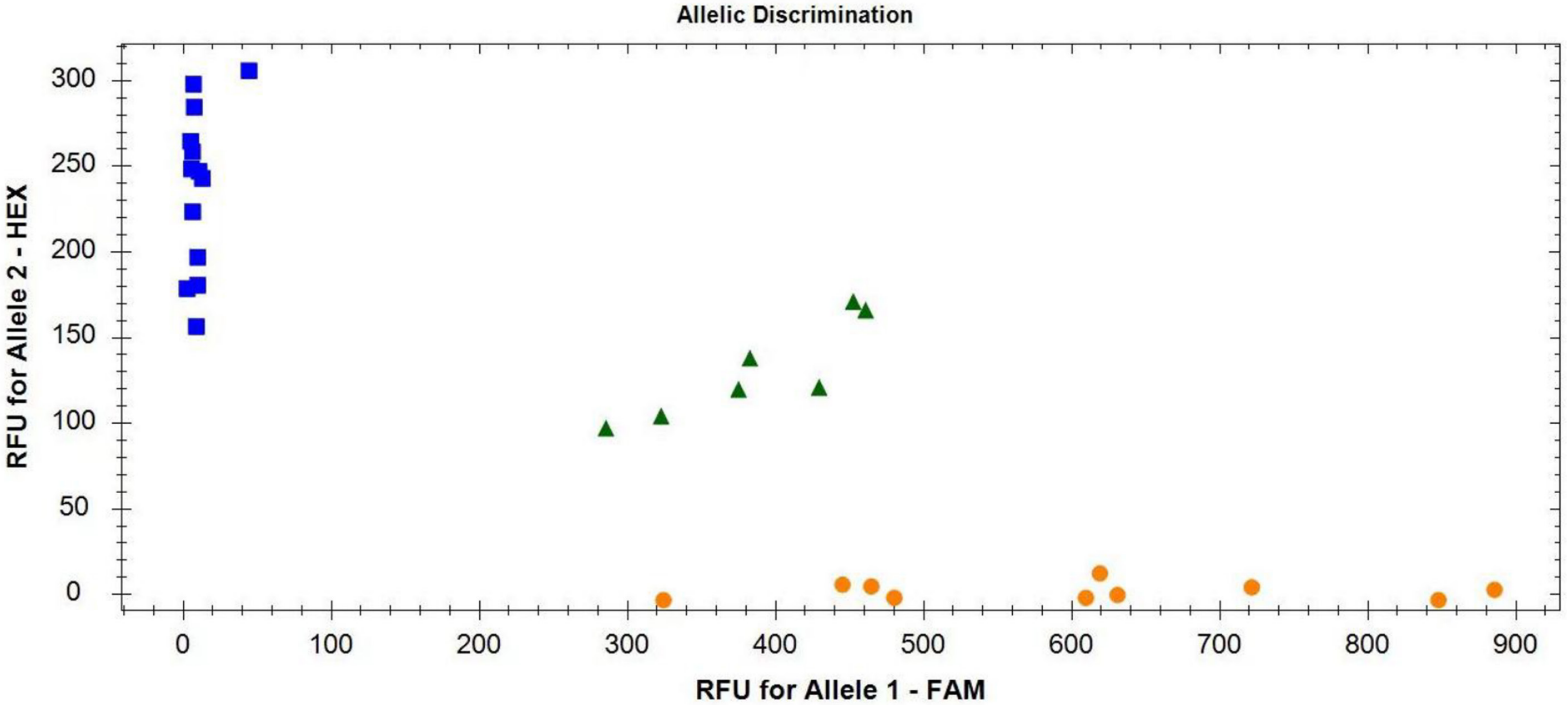
Map of genotyping detection. Instruction:□:C; △:G; ○:T.

**FIGURE 4:**
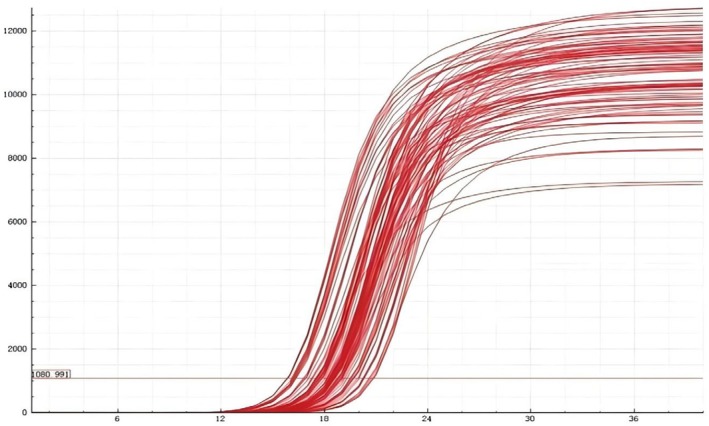
Amplification curve.

Figures 3, 4 shows that the rs1897990 and rs1524106 loci conform to the distribution patterns of genetic polymorphism typing. The rs1897990 locus has three genotypes: CT, TT, and CC. The rs1524106 locus has three genotypes: GG, GT, and TT, all of which meet the criteria for genetic polymorphism. Figure 4 displays the amplification results, which meet the quality control requirements and conditions, making them suitable for the experiment.

#### 4.3.3 Distribution of *EGF* Gene Polymorphisms rs1897990 and rs1524106 in Lung Adenocarcinoma Case and Control Groups

Table 4 shows the specimen detection results of *EGF* gene polymorphisms rs1897990 and rs1524106 in the lung adenocarcinoma case group and the healthy control group. The results indicate that the frequency of the heterozygous variant for rs1897990 differs between the two groups, and this difference is statistically significant (*p* = 0.021). In contrast, the frequency of the homozygous variant at the rs1524106 locus does not show a statistically significant difference between the two groups (*p* = 0.064).

**TABLE 4:** Polymorphic analysis of *EGF* rs1897990 and rs1524106 in lung adenocarcinoma.

Locus	Types		AB vs. AA *P*; OR (95% CI)	BB vs. AA *P*; OR (95% CI)
*EGF*	Case (AA/AB/BB)^a^	Control (AA/AB/BB)^a^		
rs1897990	20/12/10	32/5/8	**0.021**; 3.840 (1.176–12.540)	0.207; 2.00(0.676–5.917)
rs1524106	19/11/13	30/6/9	0.064; 2.895 (0.918–9.131)	0.112; 2.281(0.818–6.363)

^a^ : AA means ancestry type, AB means heterozygous mutations, BB means homozygous mutations.

Additionally, the frequencies of the homozygous variants for rs1897990 and rs1524106 do not show statistically significant differences between the two groups (*p* = 0.207; *p* = 0.112), all above detailed from Table 4.

### 4.4 Case–Control Study of the Distribution Ratios of *EGF* Gene Alleles rs1897990 and rs1524106

Table 5 shows the frequency distribution of allele genotype detection at the rs1897990 and rs1524106 loci in the lung adenocarcinoma case group and the healthy control group. The frequency of the T allele variant at the rs1897990 C > T locus differs between the two groups (case group 38.10% > control group 23.23%), and this difference is statistically significant (*p* = 0.034). The frequency of the G > T allele variant at the rs1524106 locus also shows a statistically significant difference between the two groups (case group 43.02% > control group 26.67%; *p* = 0.023).

**TABLE 5:** Distribution of rs1897990 and rs1524106 in *EGF* gene with adenocarcinoma of lung cancer.

*EGF* locus	Variable		Case group	Control group	*p*	OR (95% CI)
rs1897990	C	Allele	52 (61.90)	69 (76.67)		
	T	Allele	32 (38.10)	21 (23.23)	**0.034**	2.022 (1.048–3.903)
rs1524106	G	Allele	49 (56.98)	66 (73.33)		
	T	Allele	37 (43.02)	24 (26.67)	**0.023**	2.077 (1.033–3.910)

This suggests that the T allele variants at these two loci of the gene may represent high‐frequency adverse mutations.

### 4.5 Study of the Influence of Gender, Smoking, and Alcohol Consumption on Young Lung Adenocarcinoma Using Logistic Regression Analysis

In Table 6, univariate analysis was performed to include gender, age, smoking, alcohol consumption, and rs1897990 CT mutant, and the results showed that rs1897990 CT were statistically significant (*p* = 0.001, OR = 0.325).

**TABLE 6:** Multivariate nonconditional logistic regression analysis of factors influencing adenocarcinoma of lung.

Factors	Test	*β*	SE	*Wald*	*p*	OR
Gender	Male	−0.105	0.753	0.020	0.889	0.900
Smoking	Yes	−1.638	0.945	3.004	0.083	0.194
rs1897990	CT	−1.124	0.319	12.394	**0.001**	0.325
Alcohol	Yes	−0.780	0.939	0.690	0.406	0.458

Further logistic regression analysis incorporating gender, smoking, and alcohol consumption using SPSS software showed the results of the multifactor regression analysis, as presented in Table 6. The analysis indicated that gender, regardless of male or female groups, did not show a statistically significant difference in susceptibility to lung adenocarcinoma (*p* = 0.249, OR = 0.378), suggesting that gender may not be associated with the occurrence of young lung adenocarcinoma.

In contrast to Table 7, multivariate regression analysis showed that *EGF* rs1897990 genetic CT mutation and smoking were associated with lung adenocarcinoma in young patients (*p* = 0.043, OR = 1.309) and smoking status showed a statistically significant difference in susceptibility to lung adenocarcinoma (*p* = 0.002, OR = 2.465), indicating that smoking is a contributing factor associated with susceptibility to young lung adenocarcinoma. Alcohol consumption did not show a statistically significant difference (*p* = 4.120, OR = 0.089).

**TABLE 7:** Multivariate nonconditional logistic regression analysis of factors influencing adenocarcinoma of lung.

Factors	Test	Reference	*β*	SE	*Wald*	*p*	OR
Gender	Male	Female	−0.974	0.844	1.330	0.249	0.378
rs1897990	CT	CC	1.989	0.983	4.098	**0.043**	1.309
Smoking	Yes	No	−1.789	0.898	3.971	**0.046**	0.167
Alcohol	Yes	No	−0.697	0.849	0.674	0.412	0.498

## 5 Discussion

The increasing number of young lung cancer cases, particularly among patients under 45 years of age, poses serious health risks and creates a significant burden on society and families. Lung cancer, as a polygenic disease influenced by both genetic and environmental factors, exhibits genetic differences in its occurrence, development, treatment response, and prognosis, which are influenced by the collective action of a series of related gene SNPs on the genome. This is the result of the accumulation of many low‐effect genes. A large sample meta‐analysis [5] developed a novel lung cancer risk prediction model based on the Chinese population by analyzing the detection of 19 SNPs, which can effectively provide early warning for lung cancer.

Epidemiological studies have shown that lung cancer is a typical environmentally related disease [16, 17, 18, 19]. Smoking and environmental pollution are the main causes of lung cancer; however, studies have also found a significant increase in lung adenocarcinoma cases among nonsmokers, especially among female nonsmokers. In China, both the incidence and mortality rates of lung cancer are high, directly related to the high smoking rate, particularly among men. Although the smoking rate among men in China has been declining, it remains high (50% in 2019). The incidence of lung cancer among women in China is rising [20, 21], which may be associated with exposure to carcinogens, such as air pollution or secondhand smoke. Our logistic regression study indicates that smoking is a contributing factor associated with susceptibility to young lung adenocarcinoma, consistent with previous research. This study further demonstrates that genetic susceptibility to the *EGF* gene is associated with the occurrence of lung adenocarcinoma in younger patients, suggesting that the *EGF* gene may be a potential target for lung adenocarcinoma screening. Specifically, the two loci rs1897990 and rs1524106 of the *EGF* gene show differences in mutation frequency of the CT heterozygous variant between the two groups. The T allele of the *EGF* rs1897990 and rs1524106 loci exhibits statistically significant differences between the two groups, both of which are considered high‐risk factors.

Research data show that the *EGF* gene and its receptor gene *EGF*R are closely associated with cancer occurrence and metastasis [22, 23, 24, 25, 26, 27, 28, 29, 30, 31]. A study by Shahbazi et al. [32] indicated that *EGF* affects cancer and is related to *EGF* gene polymorphisms; for example, the G/A polymorphism at the 61 locus of the *EGF* promoter region is associated with blood *EGF* levels and susceptibility to malignant melanoma. Mutations at this locus reduce *EGF* levels and increase the risk of malignant melanoma. The findings of this study regarding the *EGF* gene rs1897990 and rs1524106 also suggest that the T allele variants at these two loci represent high‐frequency adverse mutations, increasing the risk of lung adenocarcinoma in young patients.

Currently, there have been no studies on the *EGF* gene rs1897990 and rs1524106 loci in young lung cancer populations domestically or internationally. However, there is a meta‐analysis study on the *EGF* gene and lung cancer that included six previous research results, although the loci studied differ from those in this study. Chen et al. [33] conducted a meta‐analysis examining the polymorphism of *EGF* 61A/G and its lack of correlation with lung cancer. Although the loci do not match those of this study, they pertain to the common research gene *EGF*, which may have reference and long‐term comparative significance.

This study indicates that the T allele variants of the *EGF* gene rs1897990 CT mutant were high‐frequency adverse mutations that increase the risk of lung adenocarcinoma in young patients. Smoking can promote the progression of lung cancer, and studies have shown that the interaction between smoking and the gene *PD‐L1* can synergistically promote the proliferation and progression of lung cancer cells [34]. The combined effects of smoking and genetic mutations in this study have been shown to be associated with lung adenocarcinoma in young Chinese people. Possible biological mechanisms include CT mutations that alter the structure of the *EGF* protein, affecting its ability to bind to *EGFR* and thus affect the activation of downstream signalling pathways. Alternatively, this SNP is located in the promoter region and affects the transcriptional level of *EGF*, thereby altering the activity of the *EGFR* signalling pathway.

*EGF* rs1897990 is located in the promoter or regulatory region of the *EGF* gene, which may affect transcription factor binding and thus regulate EGF expression levels. In lung adenocarcinoma, the rs1897990 C allele may enhance the activity of the *EGFR* signalling pathway and promote tumor cell survival and metastasis by upregulating *EGF*. High *EGF* levels are associated with aggressiveness and poor prognosis in a variety of cancers [35].

Rs1524106 may be located in the exon or regulatory region of the *EGF* gene, resulting in amino acid changes (such as missense mutations), affecting the structure and function of *EGF* proteins, affecting transcription efficiency or mRNA stability, and changing *EGF* expression levels.

This suggests that *EGF* polymorphisms may have potential value in the diagnosis, treatment, and even prognosis of young lung adenocarcinoma patients, but this conclusion urgently requires confirmation through larger sample sizes. The limited sample size in this study, partly attributable to the notably low prevalence of lung adenocarcinoma in younger populations, may have constrained our statistical power to identify modest but clinically meaningful associations. This epidemiological characteristic inherently restricts patient availability, particularly when focusing on age‐specific subgroups. While our findings align with existing biological mechanisms, the reduced participant numbers increase susceptibility to both Type II errors and potential selection bias, potentially limiting the external validity of these observations to broader demographics. Nevertheless, the exploratory nature of this investigation provides clinically relevant insights into a rarely studied cohort. Future multicenter collaborations aggregating data across institutions are strongly recommended to overcome recruitment challenges and substantiate these preliminary results in adequately powered analyses and more samples. Additionally, a long‐term survival follow‐up of over 3 years will be conducted with the 43 patients included in this cohort, combining the T mutation of these gene loci with smoking factors for gene‐survival prognosis interaction analysis.

## 6 Conclusion

The EGF gene variants rs1897990 and rs1524106 are significant risk factors for lung adenocarcinoma in young individuals (≤ 45 years), particularly when combined with smoking. Tobacco use appears to potentiate the oncogenic effects of these variants, further increasing susceptibility to early‐onset lung adenocarcinoma.

## Author Contributions

Huiwen Pan, Xiangyang Wang, Lijie Zheng, and Jinye Wang made contributions to the conception, design, conduct, and data acquisition. Jingfeng Zhu, Huiwen Pan, Aizhong Shao, and Guowen Ding interpreted the clinical trial, samples, experiments, and data. Huiwen Pan and Zjijie Fang wrote and edited the manuscript. All authors agreed to be personally accountable for their own contributions.

## Ethics Statement

All the experiments in this study were conducted in accordance with the relevant regulations and in accordance with the Declaration of Helsinki. This study was approved by the Ethics Committee of Jiangsu University.

## Conflicts of Interest

The authors declare no conflicts of interest.

## Supporting information


**Data S1.** Supporting information.

## Data Availability

The authors confirm that the data supporting the findings of this study are available within the article and its Supporting Information.
